# Linear models for joint association and linkage QTL mapping

**DOI:** 10.1186/1297-9686-41-43

**Published:** 2009-09-29

**Authors:** Andrés Legarra, Rohan L Fernando

**Affiliations:** 1 INRA, UR631, BP 52627, 31326 Castanet Tolosan, France; 2 Department of Animal Science, Iowa State University, Ames, IA, USA; 3 Center for Integrated Animal Genomics, Iowa State University, Ames, IA, USA

## Abstract

**Background:**

Populational linkage disequilibrium and within-family linkage are commonly used for QTL mapping and marker assisted selection. The combination of both results in more robust and accurate locations of the QTL, but models proposed so far have been either single marker, complex in practice or well fit to a particular family structure.

**Results:**

We herein present linear model theory to come up with additive effects of the QTL alleles in any member of a general pedigree, conditional to observed markers and pedigree, accounting for possible linkage disequilibrium among QTLs and markers. The model is based on association analysis in the founders; further, the additive effect of the QTLs transmitted to the descendants is a weighted (by the probabilities of transmission) average of the substitution effects of founders' haplotypes. The model allows for non-complete linkage disequilibrium QTL-markers in the founders. Two submodels are presented: a simple and easy to implement Haley-Knott type regression for half-sib families, and a general mixed (variance component) model for general pedigrees. The model can use information from all markers. The performance of the regression method is compared by simulation with a more complex IBD method by Meuwissen and Goddard. Numerical examples are provided.

**Conclusion:**

The linear model theory provides a useful framework for QTL mapping with dense marker maps. Results show similar accuracies but a bias of the IBD method towards the center of the region. Computations for the linear regression model are extremely simple, in contrast with IBD methods. Extensions of the model to genomic selection and multi-QTL mapping are straightforward.

## Background

Linkage analysis (LA) is a popular tool for QTL detection and localization. Its accuracy is limited by the number of meioses observed in the studied pedigree, which can represent several centiMorgan. Linkage disequilibrium (LD, also called gametic phase disequilibrium) is the non-random association among different loci, and is increasingly used in human and agricultural association studies for gene mapping. The joint use of LD and LA (also called LDLA) permits to map QTL more accurately than LA while retaining its robustness to spurious associations, and this technique has been applied in human [1], plant [2], and livestock [3] populations. This is achieved by explicitely modelling relatedness not accounted for in association analysis [2]. LDLA is also robust to non-additive modes of inheritance [4]. In addition, the joint use of LD and LA makes it possible to test linkage alone or linkage disequilibrium separately [1]. A characteristic of plants and livestock is that often, close pedigree relationships exist and are recorded among the individuals genotyped for QTL detection (e.g., bulls or plant varieties), and including these relationships in the analyses can be worthwhile.

In livestock, several approaches have been proposed to take into account LD information within LA [3,5,6]. These methods model the process generating LD among the putative QTL and the surrounding markers; this process can quickly become unmanageable in the general case [7], and even difficult to approximate [8-10]. Extensions of LD models to include LA (that is, the cosegregation of markers and QTL due to physical linkage) are cumbersome for the general case [6] or restricted to certain pedigree structures like half-sibs families (C. Cierco, pers. comm.). The parameters of LD generating processes can be either estimated from the data, which is often difficult, or fixed a priori which is unsatisfactory. The existence or not of these events in the past history of a population is unknown. Therefore the validity of any assumptions is largely unknown.

An alternative is QTL mapping by simple association (regression in the case of quantitative traits) of phenotypes on marker alleles, which has been s.hown to be an effective method [11,12], while retaining simplicity; this is widely used in human genetics [13]. On the other hand, QTL mapping in livestock by LA relies heavily on the use of half- and full-sibs families and relatively simple ascertainment of phases and transmission probabilities (e.g. [14]). For this reason, Haley-Knott type regressions for simple designs [14] and variance component methods for more complex designs [15] are well adapted, computationally simpler and almost as good [16,17] as full integrated likelihoods [18,19]. Linear models are appealing for their ease of use and understanding and good performance.

In this work, we combine association analysis with probabilities of transmission using conditional expectations. Ultimately, we come up with linear models for joint association and linkage mapping, which are generalizations of LA mapping. Two particular cases will be detailed: a half-sib regression which applies in many livestock practical settings, and a general mixed model approach valid for any type of pedigree.

## Methods

This section is organised as follows. In the subsection "Splitting QTL effects", we show how to come up with expectations for gametic QTL effects integrating association and linkage. The following two subsections "LDLA Haley-Knott type regression" and "Variance components mapping" explicitly present two linear models (Haley-Knott type regression for half-sib families and a general mixed model for a general pedigree) and the statistical tests that lead to QTL detection, location, and ascertainment of the hypothesis linkage, association, both or lack of both. Numerical examples and performance of the methods are illustrated by simulations in subsection "Illustrations", under two different scenarios.

### Splitting QTL effects

In this section we will show how QTL effects can be split in a part conditional on LD in the founders and cosegregation, and another part which is unconditional on LD in the founders. This results in a flexible linear model setting. Throughout the paper, we will assume a polymorphic QTL with an unknown number of alleles *nq*: {*q*_1 _⋯ *q*_*nq*_}, with effects ***α ***= (*α*_1 _⋯ *α*_*nq*_); dominance is not considered. Let **v **denote the additive effects of all gametes -carriers of QTLs- in a population; this will be referred to as "gametic effects" (e.g. [15]).

In the following we consider haplotypes, which are phased markers, i.e., a set of 1, 2, or several ordered markers on the same chromosome. Haplotypes can be classified in classes. Classes can be formed by simple classification or by more sophisticated techniques such as cluster analysis [20,21]. For the sake of discussion we will assume that haplotypes are composed of two markers with a putative QTL located at the middle, but our approach is general and conditional only on the existence of haplotype classes.

In all the following, we generally consider a single position in the genome. This position is situated on a specific chromosome number of the physical map or karyotype; for example, BTA14. In a diploid species, each individual has two copies of each chromosome: one from the paternal side and one from the maternal side. Identification of the origin of each chromosome copy is not always possible. In the following, when referring to any given chromosome pair containing a specific locus of the genome and to distinguish the two chromosome copies, we shall note them 1 and 2.

The haplotype (*j*-th chromosome in *i*-th individual, *j *= {1, 2}) can be assigned to a haplotype class *k *through a function *δ*( ) acting on a haplotype *h*. In its simplest form, *δ*( ) is a lookup table. So, for the case of two flanking SNPs, classes are 1 to 4, composed of haplotypes 00, 01, 10 and 11. The number of haplotype classes at the candidate position is *nh*.

We assume that linkage disequilibrium exists between haplotype classes and QTL alleles. Conditional on each haplotype class, population frequencies for a QTL state are denoted by matrix ***π ***= {*π*_1,1_⋯ *π*_*nq*, *nh*_}. That is, the probability of QTL state *l *conditional to haplotype class *k *is Pr(*Q *≡ *q*_*l*_|*k*) = *π*_*l*, *k*_. Assuming linkage equilibrium, *π*_*l*, 1 _= ⋯ = *π*_*l*, *nh *_= *π*_*l*_, the marginal population frequency of the *l*-th allele of the QTL. In this situation, haplotype classes are not informative on QTL states. However, given disequibrium between the markers loci and the QTL locus, *π*_*l*_, ... will vary among the different haplotype classes.

#### Founders

The haplotype of a founder individual *i *on chromosome *j *is  and belongs to a class *k *(*δ*() = *k*). The distribution of additive gametic effect  conditional on *k *is determined by ***π***:(1)

and the expectation of  conditional on the haplotype is:(2)

Neither the ***α ***effects nor the ***π ***proportions are known in practice. Thus, we propose to substitute the summation ∑*α*_*l*_*π*_*l*, *k *_by a term *β*_*k *_; that is, to substitute the weighted effects of QTL alleles for each haplotype class by the overall within-class mean. This amounts to considering *β*_*k *_as the "substitution effect", at the population level, of the haplotype. This is precisely what is done in association analysis of quantitative traits. The set of different haplotype substitution effects is ***β ***= {*β*_1_,⋯*β*_*nh*_}. In this new formulation:(3)

Now,  can be modelled as the sum of a conditional expectation plus a deviation: , where this deviation (assuming the true state of the QTL is *q*_*l*_) is  as above. The deviation  has a discrete distribution with possible states {(*α*_1 _- *β*_*k*_),⋯(*α*_*nq *_- *β*_*k*_)} with probabilities {*π*_1, *k*_,⋯ *π*_*nq*, *k*_}, which are generally unknown.

#### Non-founders

For a non-founder individual *i*, let  be the probability that the QTL allele at chromosome *j *of individual *i *is inherited from the QTL allele at chromosome *x *of its father; and let  probability that allele at chromosome *j *is inherited from the chromosome *y *of its mother. In the absence of marker information, these are 0.5. Assume that these probabilities have been computed, conditional on all marker information (**m**), using one of several methods [14,22-25]. We will refer to these probabilities as PDQ's (probability of descent for a QTL allele) [26]; they can be put together in a row vector **w**_*i*, *j *_(while each PDQ is a conditional probability, we do not explicitly include **m **in the notation for simplicity in the following expressions).

where the subscripts 1 and 2 refer to the two QTL alleles of the sire and the dam. In the expression above, four probabilities are needed because maternal and paternal origin can not always be stablished with certainty [26] and, for the same reason, labels 1 and 2 are used instead of "paternal" and "maternal" for each QTL allele in each individual. Elements in **w**_*i*, *j *_sum to 1.

The conditional distribution of , the gametic effect, is a discrete set of QTL effects ***α***, with probabilities dependent on, first, the QTL state of its parents; and second, on the probabilities of transmission of these parental QTLs towards *i*. That is:(4)

In particular, if the parents of *i *are among the founders, then it follows that:(5)

It follows that the expectation of  conditional on marker information and the rest of parameters is then simply:(6)

which, if the parents are founders, is:(7)

because of the properties of expectations (i.e., we can factor out **w**_*i*, *j*_)^. ^That is, the expected value of a gametic effect is equal to the substitution effects of the parents' haplotypes, weighted by the corresponding transmission probabilities. This is a particular case of a general, recursive formula that also works if the parents of the individual are non-founders themselves:(8)

The , the deviation of  with respect to its expectation has states  with associated probabilities  which are conditional on marker information as well.

The two building blocks in the previous section (modelling of expectations of gametic effects in founders by LD, and of non founders by conditioning on founders and LA) allow us to construct several linear models considering LD, LA, or both. In the next two sections, we will detail two linear models including LD and LA for cases commonly used in livestock genetics: a regression approach applied to idealized pedigree structures (half-sib families), and a more flexible variance component approach which can be used for general pedigree structures.

### LDLA Haley-Knott type regression

Consider *n *sires with **m **marker information. Assume further that QTL states at the sires are independent, conditional on their haplotypes and the corresponding conditional probabilities ***π ***(i.e. we assume no other relationship among sires beyond haplotype similarities, which is usual in this type of regression [14]). Suppose each of the *n *sires is mated to several dams with one daughter per dam - a half-sib design. As before, let  be the probability that the QTL allele at chromosome *j *of individual *i *is inherited from chromosome *x *of the sire; let  be the probability that the QTL allele at chromosome *j *is inherited from chromosome *y *of the dam; these PDQ's, computed based on **m**, can be put together in a matrix **W**_*i*_.

The expectation of the phenotype *y*_*i *_of a given offspring *i *from sire *s *and dam *d*, conditional on its parents' gametic effects is:(9)

Gametic effects can be split, as shown above. A part is conditional on linkage disequilibrium in the founders (*E*(*v*)), which in turn can be conditioned on haplotype substitution effects ***β ***. Another part is not conditional on linkage disequilibrium at the founders (v*). Then:(10)

Note that, in the preceding expression, we assume that haplotypes in the sire and dam are known with certainty. Assuming paternal (*p*) and maternal (*m*) origins can be established with certainty, it is possible to further simplify the expression by condensing dams' information. First, it is possible to condition only on the deviations v* in the sire, because in this design v*'s for the dams are generally difficult to estimate and non-estimable in least-squares regression. Second, we can assume that the proportions ***π ***in the founders are still accurate one generation later - that is, the decay of LD is slow, which holds for short distances (≈ 1% per generation in intervals of 1 cM). If this holds, it is possible to change the weighted substitution effect of the two haplotypes in the dam,  and , to the substitution effect of the haplotype found in the maternally inherited chromosome of descendant *i*(). This strategy was followed by Farnir et al. [5]. Then:(11)

where **w**_*s*, *i *_is a row vector with the two PDQ's from chromosomes 1 and 2 in the sire towards the paternal chromosome in *i*. Extension to *n *sires is immediate:(12)

where **W**_*p *_are the PDQ's from sires to paternal chromosome in the offspring;  is the set of "residual" gametic effects in the sires; and **Q**_*s *_and **Q**_*m *_are incidence matrices relating, haplotypes in the sires, and maternal haplotypes in the offspring, to appropriate elements in ***β***. Last, **Z**_*p *_and **Z**_*m *_are appropriate incidence matrices relating paternal and maternal gametes in the progeny to records. This conditional expectation immediately translates into a statistical model:(13)

where **e **is a vector of residuals. This model can be fitted by, for example, least-squares. Tests for QTL detection and location using interval mapping can be done by likelihood ratio or F-tests, assuming homoscedasticity of variances. Variances are indeed not homogeneous, for example, if a QTL is fixed within a haplotype class but not in another. The non consideration of dam effects also inflates the residual variance. Note, in addition, that the model is generally not full-rank:  effects are non estimable within-sire (but their contrasts are). The *β *coefficients will be estimable if they are not confounded with any  gametic effect; that is, if no haplotype class is present in one sire only. However, this does not create any problem for QTL localization and detection.

An interesting property of the model is that it is a generalization of Haley-Knott regression [14,19], which occurs if we assume linkage equilibrium among founder haplotypes. Note that spurious signals due to, for example, stratification, are unlikely in this model because there is a verification, through linkage (i.e. the PDQ's) that associated haplotypes are transmitted to the next generation and still have an effect. This breaks down spurious associations that would be observed at the founders' level.

A simplified model, which does not include the v* effects is:(14)

This expression models appropriately the cosegregation of markers and those QTL in LD with them. We call this model "LD decay" because it models appropriately the decay of initial LD existant in the founders by tracing the effect of the different segments through the pedigree with the aid of flanking markers, i.e., by linkage. However, it would not detect a QTL in the case of LE.

#### Statistical testing

Many tests are possible using the statistical model in equation (13). Usually (for example in interval mapping), several possible QTL locations are tested simultaneously or sequentially. For a particular putative QTL location, the null hypothesis is the non-segregation of alleles of the QTL having different effects. This implies that all haplotype substitution effects, as well as the v* deviations, have the same value. This amounts to a common overall mean for the data, with ***β ***= 0,  = 0. There are three alternative hypothesis depending on the existence of complete linkage disequilibrium, only linkage, or both.

The four hypothesis are:

1. *H*_0 _(null hypothesis): No cosegregation markers-QTL effects (i.e. no linkage) and no linkage disequilibrium among haplotypes-QTL: ***β ***= 0,  = 0.

2. *H*_1_: Complete linkage disequilibrium at the founders: ***β ***≠ 0,  = 0.

3. *H*_2_: Linkage equilibrium at the founders but cosegregation markers-QTL effects: ***β ***= 0,  ≠ 0.

4. *H*_3_: Incomplete linkage disequilibrium at the founders and residual cosegregation markers-QTL effects: ***β ***≠ 0,  ≠ 0

In addition, it is possible to test H_3 _against H_1 _and H_2_.

### Variance components mapping

Extension to a variance components or mixed model mapping framework [15,27,28] is possible [29,30]. As before, let **v **be the gametic effects for all the QTL gametes in the population. We will show how the first and second moments of the joint distribution of **v **can be constructed, conditional on marker information and within haplotypic classes means and variances.

Following previous notation, the following recursive equation for gametic effects holds:(15)

Each gametic effect is modelled as (i) a weighted average of the gametic effects of its ancestors (for non-founder individuals) or of haplotypic effects (for founder individuals), plus (ii) independent random variables due to mendelian sampling [15], ***ϕ***. The expression (15) potentially includes non-founder gametic effects in the progeny of non-founder animals, allowing for generality and multigenerational pedigrees.

Note that  is partitioned into founders and non-founders, and all subsequent partitioned matrices. In particular, **W **can be partitioned accordingly, so that rows tracing the origin of founder gametes from other gametes in the population are formed by 0's. Note that the setting is very similar to a genetic groups model [31]. Rules for computing the first and second moments of the distribution of the gametic effects **v **follow [29].

#### Conditional distribution of the gametic effects

##### Conditional mean for the gametic value

The development is as in previous sections. Let  be the probability that gamete  came from haplotypic class *k*. In general, for the *j*-th allele of the *i*-th individual,

For founder alleles, conditionally on the haplotype , this is simply the mean of the corresponding haplotypic class, that is , as  is 1 for *k *= *δ*() and 0 for anything else.

For non-founders, a recursive equation holds:(16)

and therefore:(17)

where **w**_*i *_is a matrix of PDQ's as before, and *s *and *d *indicate the gametes in the father and mother. From expression (15) [31]. Thus, another representation in matrix algebra is:

where (**I **- **W**)^-1 ^represents summation over all possible paths of transmission from ancestors to descendants, and  represents the expected franction of founder gametes in the descendant gametes [31]. Matrix **Q**_*f *_is an incidence matrix relating founder gametes to founder haplotypic classes. Matrix **Q **can be recursively computed using equation (16). These expressions are similar to the QTL crossbred model [32,33], save for groups for founders, which are based on haplotype classes instead of breeds.

##### Conditional variance of the gametic value

Any  gamete can in principle be traced to one or several founder populations (i.e., haplotypic classes). Had the gamete come from the haplotype class *k*, its conditional variance of the gametic effect  would be just , where , the average gametic effect in class *k*. As the number of QTL alleles and their distribution are unknown, the different  are parameters to be estimated in the model. However, the  gamete can come from several origins, each with probability ; therefore, the distribution of the gametic effect  is a mixture. Conditioning on all possible origins *k *= (1, ... *nh*),(18)

which can be expanded [29] to:(19)

where the computations of  and have been previously shown. Note that this expression reduces to the classical one [15] under linkage equilibrium.

##### Conditional covariances

As modelled here, the conditional covariance of two gametic effects depends on the event that they are identical by descent in the observed pedigree. Let  and  be two gametes, with indexes arranged so that *i *can be a descendant of *j *but not the opposite. The QTL allele at the gamete  is one of the four gametes of its parents, *s *and *d*. The conditional covariance between the gametic values  and  is then:(20)

where the covariances in the right hand side are also conditional on **m **and ***β***. This formula is the same as for the case of linkage equilibrium in the founders [15,26]. However, the variances differ due to the different haplotype origins, and the covariances will not be the same as those under linkage equilibrium.

#### Statistical model

A linear model including gametic effects is:(21)

where **X **and **Z **are incidence matrices and **b **is a vector of fixed effects. Residuals **e **are normally distributed **e**| ~ MVN(**0**, **R**), where MVN stands for multivariate normal, and **R **= **I**.

Further, assume normality for **v **(this is an approximation). Then, , where **Q **and **G **(the covariance matrix of gametic effects) are computed as above in equations (19, 20). Under this assumption of normality, the distribution of **y **is:

where **V **= **ZGZ*' ***+ **R**, and the likelihood is:(22)

Using this likelihood, Bayesian techniques or maximum likelihood techniques can be used to infer parameters of the model and location of the QTL. In particular, mixed model equations are:(23)

Note that **G**^-1 ^can be easily constructed using partitioned matrix rules [26]. These equations might not be convenient because ***β ***is found on the right hand side. An alternative formulation uses

that is, using v* = **v - Q*β ***, which has zero expectation. The mixed model equations are then [31]:(24)

Note that  enter non-trivially into **G**. For the maximum likelihood techniques, derivative-free techniques might be used with equation (22). For the Bayesian approach, albeit the "data augmentation" of gametic effects in (23) or (24) partly simplifies computations, the full posterior conditionals of ***θ ***do not have closed forms; Metropolis-Hastings might be used. Other possible simplifications are:

• Supress v* from the model in (24), i.e. **y **= **Xb **+ **ZQ*β ***+ **e**. This implicitely assumes: (i) QTL alleles are fixed within haplotype class; and (ii) transmissions are known with certainty (i.e. PDQ's are either 0 or 1). Under these two conditions, Var(v*) = 0. This might happen for very dense marker maps where markers are fully informative on QTL state and transmissions. The result is a least-squares estimator as follows:(25)

• Assume constant variances across classes and, further, that PDQ's are known with certainty. If this is the case, Var(v*) =  and standard algorithms and software (e.g., REML) can be used.

• If variances are not constant within class but each gametic effect can be asigned exactly to a class *k *(i.e. PDQ's are either 0 or 1), then its variance is . This is a mixed model with heterogeneity of variances. This assumption is similar to that by Pérez-Enciso and Varona [33].

Again, the null hypothesis is the non-segregation of QTL effects, that is, all haplotype substitution effects, as well as the v* deviations, have a null value; save that v* are now random effects. The four hypotheses are:

1. *H*_0 _(null hypothesis): No segregation of QTL effects (i.e. no linkage) and no linkage disequilibrium haplotype-QTL: .

2. *H*_1_: Complete linkage disequilibrium at the founders: .

3. *H*_2_: Linkage equilibrium: .

4. *H*_3_: Incomplete linkage disequilibrium at the founders: .

## Illustrations

### Numerical examples

We will show how the terms in both linear models are set up. Consider the pedigree and markers in Table 1. We assumed a distance of 30 cM between markers and a QTL placed at the middle. Note that, assuming few recombinations, transmissions in the pedigree are simple to follow. From this information, it can be inferred that a recombination has occurred to form the sire gamete in 6.

**Table 1:** Pedigree and markers for the numerical example

animal	dam	sire	Maternal haplotype	Paternal haplotype
1	0	0	10	01
2	0	0	11	00
3	0	0	01	11
4	1	2	10	00
5	3	2	01	11
6	3	2	01	01
7	2	5	00	11
8	2	5	00	01

#### LDLA regression

Consider sires 2 and 5 (assuming they are unrelated) and phenotypes of offspring (4 to 6 for sire 2 and 7 and 8 for sire 5). We need to set up the incidence matrix relating ***β ***to sires' haplotypes (**Q**_*s*_) and maternal-inherited haplotypes (**Q**_*m*_). Let levels 1 to 4 in ***β ***represent haplotypes 00, 01, 10, 11. Then:

Assuming chromosome origins were established with certainty, probabilities of transmission are 0.98 for the non-recombinant and 0.02 for the recombinants (actually, double recombinants) if markers were transmitted together, or 0.5 if they were not. The matrix of PDQ's **W**_*p *_is thus:

There are four (twice the number of sires) gametic sire effects . Last, **Z**_*p *_and **Z**_*m *_are 5 × 5 identity matrices for records of individuals 4 to 8. Note that animal 5 is in the analysis both as sire and as offspring. The final equations (13) are thus:

#### Variance components mapping

In order to construct the mixed model equations we assume certain values for the class substitution effects *β' *= [0.9, 0.5, 0.5, 0.1] and for the within-class variances  = (0.09, 0.25, 0.25, 0.09) (in practice these values have to be estimated).

##### Expectation of gametic effects

Setting up the matrix **Q **for the founders implies just setting the element corresponding to the *j*-th haplotype of the *i*-th founder and the *δ *() class to 1, and all other to zero. Gametic effects are ordered within each animal. Then the first six rows of **Q **are:

where the first two rows correspond to animal 1, the next two to animal 2, and so on. Let's take non-founder animal 4. Its rows in **Q **are the product of the corresponding PDQ's times the rows in **Q **corresponding to their parents 2 (sire) and 1 (dam). That is:

The process is repeated for every individual. Individual 7 is descendant of two non-founders (sire is 5 and dam is 4), but the same logic applies.

Matrix **Q **is then:

##### Covariance matrix of gametic effects

To compute the variance we apply (19). For founders, variances are  for the first gamete in 1,  for the second,  for the first gamete in 2, and so on. For non-founders, let consider for example gamete 2 in individual 4 and gamete 2 in individual 6. Note that the terms  are contained in matrix **Q **above. If we apply the formula and ignore null terms (those  = 0):

and

We can see that the higher uncertainty in the origin of  results in a higher variance. As for the covariances, these were computed using the algorithm of Wang et al. [26]. The final covariance matrix **G **is:

#### Simulations

##### Scenarios

First, four simulations were carried out to check the behaviour of the different methods for fine mapping. We used the LDSO software for the simulations (F. Ytournel, pers. comm), a set of programs developed at INRA (T. Druet, F. Guillaume, pers. comm.) for phase determination and computation of PDQs, and user-written programs for setting up and solving the linear models.

The first set of scenarios will be termed as "drift". Two sub-scenarios differing on the size of the region of interest (5 or 20 cM) were designed. A 5 (alternatively, 20) cM region with 21 SNP markers (i.e., 20 brackets), with a biallelic QTL at position 2.125 (alternatively, 8.5) cM (at the middle of the 9th bracket). The QTL was biallelic with an effect of 1 for the second allele. No foundational event was assumed (i.e., marker and QTL alleles were assigned at random in the ancestral population). SNP alleles were assigned at random in the founders. This population evolved during 100 generations with an effective size of 100. Therefore the only source of LD was drift. After these populational events, a daughter design was simulated, with 15 sires each with 20 daughters. Phenotypes were simulated according to the QTL effects and to a residual variance of 1; no polygenic effects were simulated. This is a scenario where IBD methods are likely to perform well. Although the design is fairly small for dairy cattle, it is not unlikely for swine or sheep, and our purpose was not to provide a large amount of information.

The second two scenarios ("admixture") are radically different and include strong admixture. Again, 5 and 20 cM region are considered, with same positions for the QTL. Initially, two breeds existed differing in their polygenic average by 1. A QTL is considered with equal frequency in each breed, with an effect of 1 for the second allele. SNP alleles were assigned at random in the founders. Both breeds were crossed and a mixed population of 50 individuals evolved during 20 generations. A daughter design as before was simulated. Phenotypes were simulated according to the QTL, the inherited polygenic part of each breed, and a residual variance of 1. This scenario might generate admixture by drift if one SNP locus is indicative of breed origin.

##### Methods

We compared the performances of five different methods: (1) LA: Haley-Knott linkage analysis [14], (2) LDLA: the regression LDLA method in this work (equation 13), (3) LD decay: LDLA regression by equation (14), that is, ignoring the v* terms, (4) two-marker: regression on two-marker haplotypes (i.e., association analysis), and (5) an IBD method [3,34], which computes IBD among founders based on all markers (Lee, pers. comm.).

The simplest approach is to perform single marker association analysis, which has been shown to be as good as more complex methods in quite a variety of scenarios [35]. We nevertheless discarded this option because the simulation method places the QTL in the middle of a bracket. This automatically penalizes the single-marker analysis. Further, by using always two markers we can compare all methods in the same grounds (except IBD).

For the two-marker and IBD method, phases were assumed to be known with certainty; this might have resulted in slightly optimistic results. Performance of the different methods was based on the average error (i.e., the bias, in cM) and the mean square error (MSE, in cM^2^). All simulated populations had a minor allele frequency of 0.1 at least for the QTL. One hundred replicates were run.

## Results

Tables 2 and 3 show the results of the simulations for the "drift" scenario and Tables 4 and 5 for the "admixture" scenario.

**Table 2:** Performance of five fine-mapping methods in drift and a 5 cM segment.

Method	Bias		MSE	
LA	0.29	(0.15)	2.22	(0.22)
LDLA	0.06	(0.08)	0.67	(0.09)
LD decay	0.11	(0.08)	0.69	(0.10)
Two-marker	0.13	(0.08)	0.66	(0.10)
IBD	0.34	(0.08)	0.78	(0.15)

Bias and mean square error (in cM and cM^2^) (with standard errors) of five fine-mapping methods: linkage analysis, joint association and linkage analysis, linkage disequilibrium decay, two-marker association, and an IBD method. The scenario is drift and a 5 cM segment spanned with 21 SNP markers.

**Table 3:** Performance of five fine-mapping methods in drift and a 20 cM segment.

Method	Bias		MSE	
LA	0.51	(0.44)	19.61	(2.89)
LDLA	-0.18	(0.26)	7.06	(1.65)
LD decay	-0.12	(0.24)	5.68	(1.52)
Two-marker	-0.05	(0.24)	5.89	(1.41)
IBD	1.20	(0.19)	5.14	(1.51)

Bias and mean square error (in cM and cM^2^) (with standard errors) of five fine-mapping methods: linkage analysis, joint association and linkage analysis, linkage disequilibrium decay, two-marker association, and an IBD method. The scenario is drift and a 20 cM segment spanned with 21 SNP markers.

**Table 4:** Performance of five fine-mapping methods in admixture and a 5 cM segment.

Method	Bias		MSE	
LA	0.42	(0.14)	2.06	(0.21)
LDLA	0.31	(0.14)	2.15	(0.23)
LD decay	0.31	(0.14)	2.02	(0.21)
Two-marker	0.16	(0.13)	1.82	(0.18)
IBD	0.23	(0.13)	1.69	(0.20)

Bias and mean square error (in cM and cM^2^) (with standard errors) of five fine-mapping methods: linkage analysis, joint association and linkage analysis, linkage disequilibrium decay, two-marker association, and an IBD method. The scenario is admixture and a 5 cM segment spanned with 21 SNP markers.

**Table 5:** Performance of five fine-mapping methods in admixture and a 20 cM segment.

Method	Bias		MSE	
LA	0.58	(0.60)	36.60	(3.42)
LDLA	0.97	(0.53)	28.43	(3.06)
LD decay	0.04	(0.49)	23.76	(2.56)
Two-marker	0.17	(0.51)	25.89	(2.50)
IBD	1.94	(0.50)	28.78	(3.43)

Bias and mean square error (in cM and cM^2^) (with standard errors) of five fine-mapping methods: linkage analysis, joint association and linkage analysis, linkage disequilibrium decay, two-marker association, and an IBD method. The scenario is admixture and a 20 cM segment spanned with 21 SNP markers.

In the "drift" scenario, LA and IBD methods are biased for the 5 cM scenario, and the IBD method is biased for the 20 cM scenario. The ranking of the methods changes with the scenario, with LA being always the worst in accuracy, as expected. The reason of the inability of LA to map the QTL is simple: in small intervals, recombinations - which are needed for LA to map a QTL-seldom occur. Thus, in the 5 cM scenario, the performance of LA is roughly equivalent to random mapping of the QTL. For the remaining methods, differences are indeed largely non-significant except for the bias.

**Figure 1:**
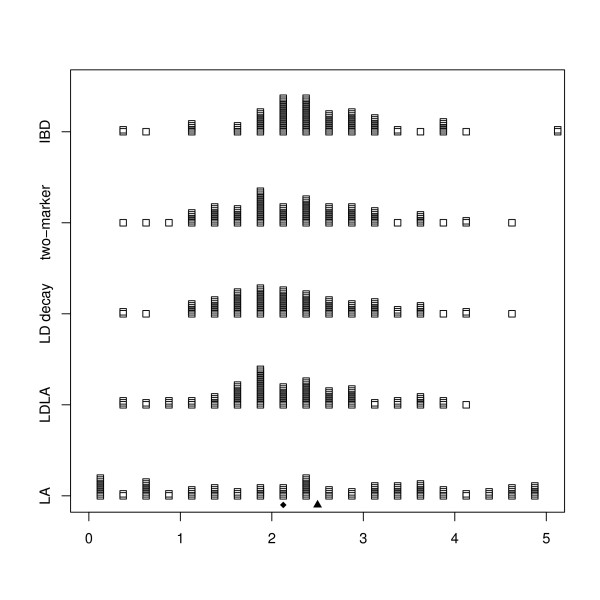
**Errors of five fine-mapping methods in drift and a 5 cM segment**. Errors (in cM) in location of the QTL by the different methods - drift and 5 cM scenario. The small triangle is the center of the segment; the small diamond is the QTL location.

Figure 1 shows a plot of estimated locations in the 100 simulations vs the QTL position in the "drift 5 cM" scenario. From the graph, it is clear that the IBD method tends towards the center of the haplotype, whereas the other methods are the less biased. The LDLA method is somewhere in the middle.

**Figure 2:**
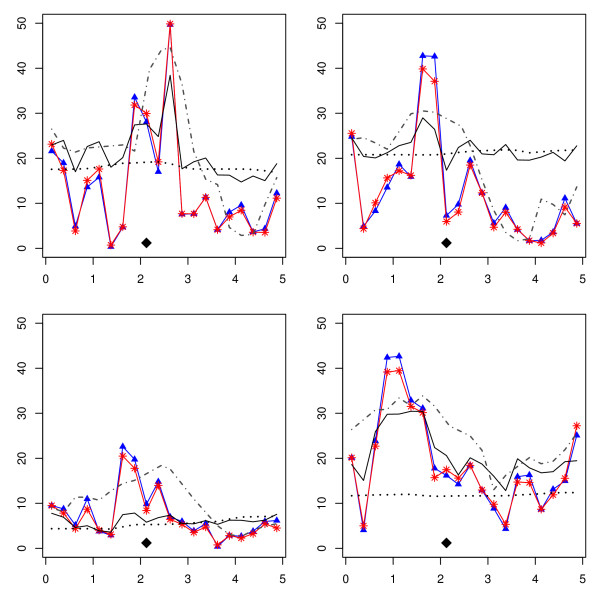
**Interval mapping profiles in the drift and 5-cM scenario**. Interval mapping profiles (minus log of the p-value) in four replicates of the drift and 5-cM scenario. LA: dotted line. LDLA: continuous line. LD decay: red, stars. Two-marker: blue, triangles. IBD: grey dot-dash line. A diamond indicates the QTL location.

Figure 2 shows the interval mapping profile of the p-value along the chromosome for four replicates of the "drift 5 cM" scenario. It can be seen that the signal of association (i.e. two-marker) is considerably smoothed by the LDLA and IBD methods; this might compromise detection power (not addressed here). It is also apparent that the IBD methods look like a weighted average of signals of close markers; this results in smoothing but also in uncertainty.

Most of these results are due to the multimarker nature, in this order, of LA, IBD and LDLA, which might tend to point central regions since, in these, haplotypes are more informative and PDQ's are better calculated. This is alleviated in the LDLA method and LD decay method by the implicit two-marker association analysis.

As for the admixture scenario, Table 4 shows basically that QTL location cannot be accurately estimated. The reason is that the scenario is not informative enough due to the low number of historical recombinations and the noise added by admixture. Differences in performances (MSE) of the different methods are not statistically significant; however, LA, LDLA and LD decay do show some bias.

**Figure 3:**
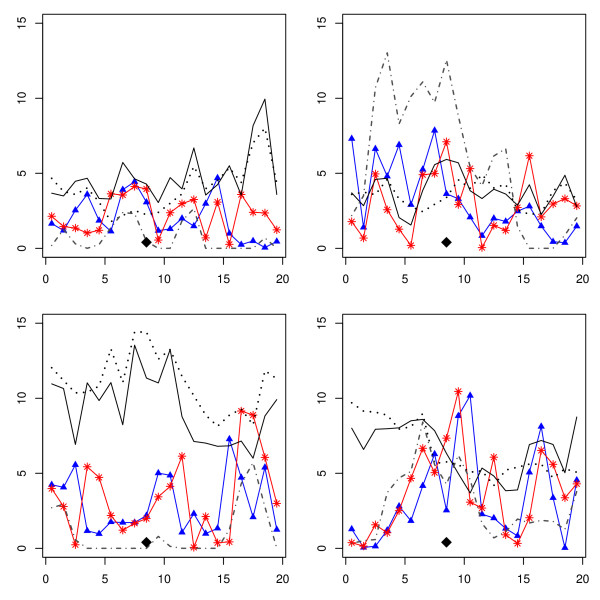
**Interval mapping profiles in the drift and 20-cM scenario**. Interval mapping profiles (minus log of the p-value) in four replicates of the admixture and 20-cM scenario. LA: dotted line. LDLA: continuous line. LD decay: red, stars. Two-marker: blue, triangles. IBD: grey dot-dash line. A diamond indicates the QTL location.

The 20 cM admixture scenario (Table 5) shows much worse performance of the mapping methods than in the drift scenario; and this, for the same reasons as above: few historical recombinations and noise added by admixture. LA is the worst method in terms of MSE, whereas the LD decay method is the best. However, differences are not significant, and no clear conclusions can be drawn. The profiles in Figure 3 are indeed very chaotic, and they would be difficult to interpret in real-life experiments.

## Discussion

### Comparison to other models for LDLA

We have presented a method for joint association and linkage, which belongs to a more general class of joint linkage disequilibrium and linkage analysis. In fact, existing methods belong to one of two exclusive classes: those that model somehow the LD phenomena and those that do not.

Some models [5,6] assumed a mutation followed by expansion of the mutant haplotype. The pertinency of this scenario in general agricultural populations (and indeed in complex diseases in humans) is arguable. The likelihood in Farnir et al. [5] was an approximation, based on the assumption of independence among contiguous markers; and the form of the likelihood was only appropriate for family designs. The more complex model in Pérez-Enciso [6] holds for any pedigree structure below the founders, but computations were difficult.

Models for association and linkage in human populations exist [1,36]. These, although very similar to our approach, are difficult to apply to livestock since they are rigidly family-structured; in addition, the QTDT [1] uses unilocus information only for transmission events, whereas in our model it is possible -- and recommended -- to use multilocus information to compute the PDQ's, and it is possible (but perhaps not useful) to define haplotype classes spanning several loci. Conversely, the QTDT has no need of PDQ calculation or - possibly - map ordering of SNPs.

The most popular model for LDLA QTL detection in livestock has been reported by Meuwissen et al. [3] and has been fairly used [20,37]. The method relies on the construction of a matrix of covariances among founders (the so-called IBD probabilities), say **H**, based on identity of state among markers; these IBD probabilities are derived following approximate coalescent models [8,9,38,39]. IBD methods use the same parameter (the variance assigned to the QTL) for both covariance due to association and covariance due to linkage. Modelling linkage and association using different parameters (*β *and *v*) allows for a greater flexibility in our model. This can be explained as follows.

### Relationship of the IBD method for LDLA to our approach

Suppose we have two marker loci flanking a QTL. Assume that LD is generated by some random process such as drift or mutation. Then, given this LD generation (LG) event, the expected value of the effect of gamete *j *for a founder individual *i *is denoted by(26)

For SNP markers, there will be four possible values for the haplotypes. Let ***β ***denote the vector of the four *β*_*k *_variables. In our approach, ***β ***is treated as a fixed effect. However, over LG events, ***β ***can be thought of as random. Suppose the LG process is such that the expected value of ***β ***over LG events is(27)

and the covariance matrix of ***β ***over LG events is(28)

The matrix **Σ **will depend of the LG process, and in the IBD method of Meuwissen and Goddard [8,9], it is the matrix of IBD probabilities at the QTL conditional on the observed marker data. Thus, when marginalized over the LG events, the mean and variance of *β*_*k *_do not depend on the marker haplotype. Similarly, the marginal (or unconditional) variance of  does not depend on the marker haplotype, and it is denoted by  It follows that the unconditional variance of

is

Now, using notation in our paper, the covariance matrix of gametic effects of the founders can be written as(29)

The covariance matrix for the entire vector of gametic effects can be computed, recursively, using equation (20) in our paper, starting with the covariance matrix in equation (29). If there is no LD,  will be zero and (29) will reduce to the , which is the covariance matrix under LE. Also, (29) depends on two variance components that relate to the gametic variance due to LD and the remainder. In the IBD method [8,9], Var(***v***_*f*_) is written as , where ***H ***is an IBD matrix with diagonals equal to 1 and off-diagonals given by . Thus, in the IBD method [8,9] the partitioning of the gametic variance due to LD and the remainder is entirely dependent of the assumptions underlying the computation of **Σ**.

A practical problem using IBD methods (our experience is with the IBD methods [3]), is that often matrix **H **turns out to be negative definite; hence the likelihood of the phenotypes is undefined. The reason is that construction of **H **is not based on a joint distribution for all founder chromosomes, but it is computed for two haplotypes (or chromosomes) at a time, marginalizing over the rest. This leads to approximate marginal probabilities in **H **instead of a joint distribution. Thus, the estimated **H **matrix is at best an approximation. A way to deal with non-positive definiteness is bending [40], or clustering (a data reduction technique) [20]. Both approaches might result in a loss of information, have unknown statistical properties and are subject to arbitrary tuning parameters.

At any rate, both modelling the LD phenomena and IBD based models rely in assumed population events. The robustness of these methods to, for example, admixtured breeds, is largely unknown and difficult to verify. Our model and those by Fernando et al. and Gilbert et al. [30,41] do not model the process generating LD among QTL and markers, and therefore are more general. The only strong assumption that they made was that of a biallelic QTL, which is overcome in ours, at the price of a greater number of unknowns.

### Originality

The originality of our approach is that (i) it is feasible and well-taylored for some agricultural populations, in particular livestock (because it relies on phase and transmission information easily ascertainable, and holds for any family structure) and corn (where indeed a similar idea -- nested association -- has been developed [42]), (ii) it is a linear model (with all the adequate machinery), while (iii) at the same time providing, based on expectations and covariances, a simple and coherent linear-models framework for association and linkage and (iv) reduces to well-known models on the hypothesis of LE or complete LD. Indeed, our models allows us to test the four relevant hypotheses (disequilibrium, linkage, both or none) and reduces to association or linkage under the respective hypothesis, which is not the case for other methods such as IBD models for example, which assume that LD exists.

Our method is computationally simple to use, provided that phases and PDQ's can be accurately calculated. If this is not the case, inference is possible, in principle, by integrating over all the joint distribution of phases and transmissions. After phase determination and computation of PDQs, all the machinery of the linear models can be applied. This makes it possible to include simultaneously other effects (environmental effects, polygenic effects) and the use of other tools such as permutation tests, bootstrapping and in particular the simultaneous fit of several QTLs [43]. The latter one is of particular interest for recent developments in genome-wide genetic evaluation ("genomic selection") using LDLA. For example, the number of simultaneous effects fitted by Calus et al. [44] was ~ 600,000 for two-marker haplotypes in a genome composed of ~ 2300 markers. If a "LD decay model" is used (such as equation 25) the number of equations is linear in the number of loci, while retaining the use of LD and of some of the LA. Even with the full linkage and association model (equation 24), sparsity of the mixed model equations is guaranteed.

A practical problem with the method is how to define "classes" of haplotypes; for example, how many markers to include in the definition of the classes. Including more markers in the definition of the haplotype is straightforward, but probably at the price of greater complexity. The optimal number of markers seems scenario dependent [12,35]. A practical rule of thumb is to define classes that are manageable - that is, not too many. For example, Druet et al. [45] considered haplotypes spanning either 3 or 10 markers, with a number of classes of 8 and 700, respectively. The latter were too many and had to be clustered. They observed that 3-marker haplotypes provided narrower intervals than 10-marker haplotypes, at the possible price of more false-positive detections. With multiallelic markers the two-loci classes might be impractical. Two options might be (i) to consider the closest microsatellite, or (ii) to split the effect of a haplotype class in a sum of individual marker locus effects. In this option a descendant of haplotype, say, "13" with probability *w *would be in expectation *w *times the effect of allele 1 at the first locus, plus *w *times the effect of allele 3 at the second locus.

### Performance of the method

Computations for any of the regression methods (LA, LDLA, LD decay and two-marker) were extremely fast. For the case of LDLA, computing one position took 0.02 seconds. For the IBD method, each position took about 40 seconds.

Results show no clear ranking of methods. Indeed, the fact that the IBD method is often biased deserves further attention for small chromosomal segments, albeit its good performance in the drift 20-cM simulation shows the value of multi-marker information in relatively sparse maps. The LD decay method is possibly the best across all scenarios, but the two-marker regression analysis is almost as good. Zhao et al. [35] have shown that the even simpler method of single-marker regression performed slightly better than two-marker regression. Thus, future work should compare our methods (LDLA or LD decay) with single-marker association.

The admixture simulation shows basically that the extra noise generated affected all methods for localization of QTLs; whether this holds for detection remains to be seen. Thus, more extensive simulations need to be undertaken to compare accuracy, power, and robustness to spurious associations of the different methods.

It seems, nevertheless, that our linear model (LDLA or LD decay) is at least as good in performance as the IBD method, while keeping simplicity. In fact, for small chromosomal segments, association between QTL and markers is very informative [12,39]. As an aside, simulations should not place the QTL at the center of the segment as this hides bias of the methods and artificially decreases MSE.

It is expected that, for narrower and narrower marker intervals, most information will be captured by the LD term and less by the LA terms. At the limit, if the QTL *is *the marker, the variance for the gametic effect *v *(v*) will be null and all information will be contained in ***β***. On the other hand, for very distant markers, variance of *v *will be high and ***β ***will tend to zero. Still, linkage will still be used in modelling the pedigree transmission of fully associated marker effects.

## Conclusion

We have presented simple linear models for QTL detection and localization including populational linkage disequilibrium and within-family cosegregation. The methods uses all available information (i.e., multiple markers and pedigrees). The performance of these methods is satisfactory, as shown by simulations. These methods are computationally much simpler than other proposals. Extensions to multiple QTL mapping and genomic selection are straightforward. These methods should help researchers in QTL mapping and marker assisted selection, in particular in livestock species, where the required information is available, just like regression is more used than full-likelihood methods [14], when possible.

## Competing interests

The authors declare that they have no competing interests.

## Authors' contributions

AL and RF derived the theory and wrote the text. AL performed the simulations and the numerical examples.
